# Towards multi-order hard X-ray imaging with multilayer zone plates

**DOI:** 10.1107/S1600576714026016

**Published:** 2015-01-30

**Authors:** Markus Osterhoff, Christian Eberl, Florian Döring, Robin N. Wilke, Jesper Wallentin, Hans-Ulrich Krebs, Michael Sprung, Tim Salditt

**Affiliations:** a Institut für Röntgenphysik, Georg-August-Universität Göttingen, Friedrich-Hund-Platz 1, 37077 Göttingen, Germany; b Institut für Materialphysik, Georg-August-Universität Göttingen, Friedrich-Hund-Platz 1, 37077 Göttingen, Germany; c DESY Photon Science, Notkestrasse 85, 22607 Hamburg, Germany

**Keywords:** X-ray imaging, multilayer zone plates

## Abstract

This article describes holographic imaging experiments using a hard X-ray multilayer zone plate (MZP) with an outermost zone width of 10 nm at a photon energy of 18 keV. An order-sorting aperture (OSA) is omitted and emulated during data analysis by a ‘software OSA’. Scanning transmission X-ray microscopy usually carried out in the focal plane is generalized to the holographic regime. The MZP focus is characterized by a three-plane phase-retrieval algorithm to an FWHM of 10 nm.

## Abstract 2

Multilayer zone plates can be used for holographic imaging without an order-sorting aperture.

## 1. Introduction   

Coherent X-ray imaging has emerged as an active field of research, enabling ambient-conditions investigations of untreated biological and non-organic specimens with a few nanometres resolution (Paganin, 2006 ▶; Nugent, 2010 ▶; Rehbein *et al.*, 2009 ▶). Such imaging experiments yield quantitative contrast by phase reconstruction (Pfeiffer *et al.*, 2006 ▶), giving access to the electron or mass density in two or three dimensions (Bartels *et al.*, 2012 ▶; Holler *et al.*, 2012 ▶). While algorithms like ptychography have overcome the limitation of the resolution by the focal spot size (Thibault *et al.*, 2008 ▶), a direct probing with small beams of a few nanometres is still required for contrast mechanisms like nanodiffraction (Hruszkewycz *et al.*, 2014 ▶; Weinhausen *et al.*, 2014 ▶) and fluorescence mapping (de Jonge & Vogt, 2010 ▶), or to use the X-ray beam for local excitation as in X-ray beam induced current studies (Vyvenko *et al.*, 2002 ▶).

With the 10 nm ‘barrier’ passed (Mimura *et al.*, 2010 ▶), it was only a matter of time until two-dimensional focusing below 5 nm could be achieved using a multilayer zone plate (MZP) (Döring *et al.*, 2013 ▶; Osterhoff *et al.*, 2013 ▶). Here we present current experiments on our route towards nanoscale imaging using a multi-order MZP illumination. Since the positioning and alignment of order-sorting apertures (OSAs) to suppress negative and higher orders of the diffractive element (i) leads to additional background scattering, (ii) has to be performed and checked and (iii) is extremely challenging for small focal distances 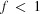
 mm, we investigate if the OSA can be replaced by image processing and analysis of far-field patterns.

This paper is organized as follows. In §[Sec sec2]2, we describe and quantify our focusing experiment. Results based on holographic recordings are presented and discussed in §[Sec sec3]3. In §[Sec sec4]4, a generalization of scanning transmission X-ray microscopy (STXM) to the holographic regime (holo-STXM) with the sample in the defocus is presented. For comprehension, two experimental data sets and analysis code are available online as supporting information. 1

## 2. Focusing experiment   

### 2.1. Advantage of combined optics   

A key challenge in X-ray optics is the fabrication of large-aperture focusing devices of high efficiency that take a coherent part of the incoming synchrotron radiation beam and focus it down to a small beam investigating the specimen. The aperture 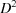 of such an optic should accept the coherence area 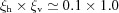
 mm of the synchrotron beam. This size is easily matchable by optics using total reflection or multilayer mirrors in grazing-incidence geometry. Compound refractive lenses (CRLs) (Snigirev *et al.*, 1996 ▶; Schroer & Lengeler, 2005 ▶) and traditional Fresnel zone plates (FZPs) (Niemann *et al.*, 1974 ▶; Chao *et al.*, 2009 ▶) also reach these sizes. The lithographic fabrication of FZPs allows for feature sizes down to 20 nm and optical thicknesses below 1 µm (Vila-Comamala *et al.*, 2011 ▶). FZPs are routinely used for soft X-ray microscopes with resolutions down to 10 nm (Chao *et al.*, 2012 ▶) and can now be used up to 12 keV (Jefimovs *et al.*, 2007 ▶). For the photon energy range 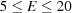
 keV, Kirkpatrick–Baez (KB) (Kirkpatrick & Baez, 1948 ▶) mirror systems in total reflection mode or with multilayer coatings (Morawe & Osterhoff, 2010 ▶) are used; by design, focal lengths 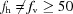
 mm. CRLs are well suited focusing devices even for higher energies above 20 keV.

To extend the spectral range of diffractive optics, multilayer zone plates (Saitoh *et al.*, 1989 ▶) and one-dimensional multilayer Laue lenses (Yan *et al.*, 2013 ▶) with outermost zone widths 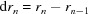 of a few nanometres and optical thicknesses of several micrometres are used. The layer radii 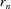 are determined by the Fresnel zone plate law, 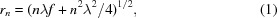
with wavelength λ. Assuming λ = 0.1 nm, *f* = 1 mm, and requesting a resolution given by the outermost zone width 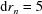
 nm, the aperture is only 20 µm, ignoring a significant fraction of the beam. A combined scheme prefocuses the incoming beam to the MZP and can achieve two-dimensional spots of 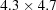
 nm at 7.9 keV and slightly larger at 13.8 keV (Döring *et al.*, 2013 ▶; Osterhoff *et al.*, 2013 ▶).

### 2.2. MZP design and fabrication   

**Figure 1:**
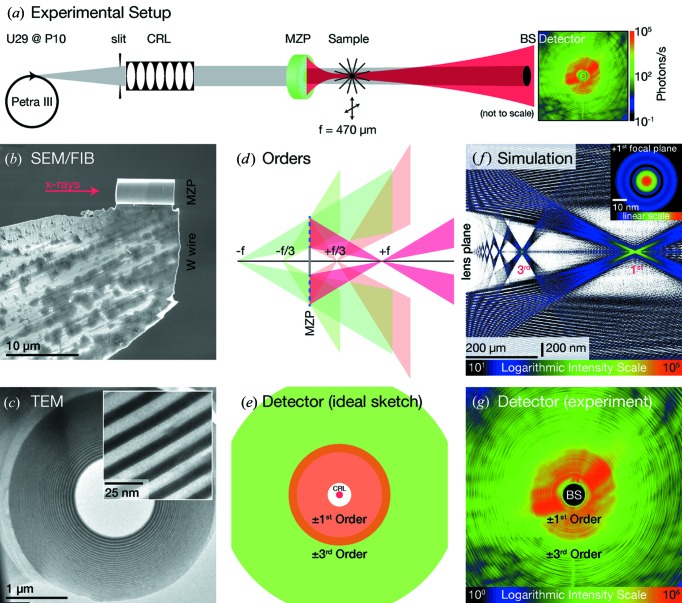
Experimental setup of the MZP imaging experiments. (*a*) The synchrotron radiation beam is prefocused by 18 beryllium CRLs and then focused down to about 10 nm using an MZP, with the sample in the focus or defocus. The zeroth order is absorbed by a beamstop (BS). (*b*) SEM image taken during FIB preparation of an MZP (side view) mounted on a W tip. (*c*) Transmission electron micrograph of a lamella cut (equivalent to the lens). (*d*) Sketch showing the different orders from a binary zone plate. (*e*) Sketch of an idealized detector image, showing the central CRL beam and the first four orders. (*f*) Simulation of the focused intensity, in a logarithmic false-colour representation, along the optical axis; compare to (*d*). (*g*) Typical far-field diffraction patterns of the MZP, logarithmic intensity scale; compare to (*e*).

For the experiments sketched in Fig. 1 ▶(*a*), an MZP consisting of 69 alternating thin layers of ZrO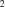 and Ta
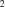
O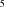 has been fabricated using both pulsed laser deposition (PLD) and a focused ion beam (FIB). The layers are deposited onto a rotating pulled glass wire with diameter 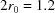
 µm; the layers closely follow the zone plate law [equation (1)] from 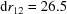
 nm to 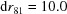
 nm and give a circular aperture of 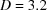
 µm with a focal length of 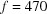
 µm. The optical thickness is 8.9 µm. For more details on the PLD process, see Eberl *et al.* (2014 ▶), and references therein.

Fig. 1 ▶(*b*) shows a scanning electron microscopy (SEM) image of the MZP during FIB preparation (side view), after the lens has been glued to a W tip. In Fig. 1 ▶(*c*), a micrograph of a transmission electron microscopy (TEM) lamella (not the lens itself) is shown; the inset shows part of the smallest zones. The PLD process does not suffer from cumulative roughness, yielding very precise alternating layers following equation (1).

### 2.3. Experimental setup   

The focusing and imaging experiments presented in this paper were carried out at the GINIX endstation (Kalbfleisch *et al.*, 2011 ▶) of the PETRA III coherence beamline P10, DESY, Hamburg (Germany); see Fig. 1 ▶(*a*) for a sketch of the relevant components. The photons of energy 18 keV were pre-focused by a stack of 18 Be CRLs to a size of 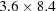
 µm, as determined by knife-edge scans. Two beam stops (BSs) were mounted to protect the Pilatus 300k single-photon-counting pixelated detector (Dectris Inc., Baden, Switzerland), mounted 5.1 m downstream of the focus, from the intense primary beam.

Note that the MZP was used without a pinhole and without an OSA; apart from the nominal first-order focus at 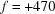
 µm, higher odd-numbered orders at 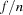 and negative orders at 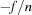 exist; see Fig. 1 ▶(*d*) for a sketch. On the detector, different orders *n* can be roughly separated in position because they arise from different cone beams; see Fig. 1 ▶(*e*) for an idealized sketch and Fig. 1 ▶(*g*) for a typical detector image. However, positive and negative orders for the same *n* fall into the same regions. Below, we investigate whether a ‘software OSA’ can be used to distinguish between positive and negative orders.

The multi-focus along the optical axis is illustrated by a longitudinal wave-optical simulation, as shown in Fig. 1 ▶(*f*); the nominal focus size of 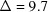
 nm (FWHM) can be calculated from a wave-optical simulation in the first-order focal plane (see the inset). Knife-edge scans to directly determine the focal spot size turned out to be impractical owing to the long penetration depth of X-rays at 18 keV and the required smoothness and angular alignment of the sample.

The focusing efficiency of the MZP was measured using the Pilatus detector and sets of Mo foils for attenuation to be 2.0% per positive and negative first order; taking the geometrical acceptance and the prefocusing into account, the +1st-order focusing efficiency of the active zones is estimated to be 6.9%, with a flux of 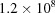
 photons per second.

### 2.4. Focus reconstruction   

From far-field intensity measurements, the complex valued optical field in the focal plane can be reconstructed if suitable *a priori* knowledge is provided to common phase reconstruction algorithms (Fienup, 1982 ▶; Bauschke *et al.*, 2002 ▶; Luke, 2005 ▶). Such constraints usually are

(1) finite support, *i.e.* vanishing electron density outside a bounded region in the sample plane,

(2) flat phase, *i.e.* a real-valued transmission function and real-valued illumination,

(3) overlap, *i.e.* a ptychographic scan with a step size smaller than the beam size.

Phase reconstruction algorithms like Gerchberg–Saxton or hybrid input–output numerically propagate the complex field between detector plane 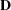 and sample plane 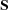 (Gerchberg & Saxton, 1972 ▶). In 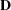, the phase information is kept, but the amplitude is re-set to match the measured intensity. In 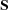, in the case of a finite support, the amplitude and phase outside a bounded region are each set to a constant. The back-and-forth propagation and constraints are repeated, iteratively reconstructing the phase in 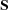.

For MZP focal fields 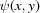, this scheme is extended to three planes (Quiney *et al.*, 2006 ▶). The detector plane 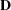 and the focal plane 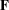 are numerically connected by a Fourier transformation 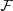; 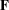 and the lens plane 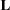 are connected by a Fresnel near-field propagator 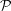 (Schmidt, 2010 ▶):
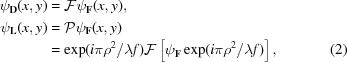
with 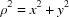. The propagator (2) adapts the pixel size 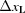 in the lens plane according to 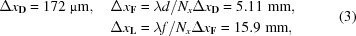
where 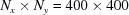 pixels have been used and the focus–detector distance is 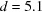
 m. The propagations 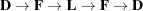 are complemented by the following constraints:

(i) 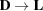: the field outside the lens area of diameter *D* = 3.2 µm is considered undisturbed, *i.e.* homogeneous phase and intensity.

(ii) 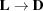: the propagated phases are kept, the amplitudes are set to the square root of measured intensities per pixel.

**Figure 2:**
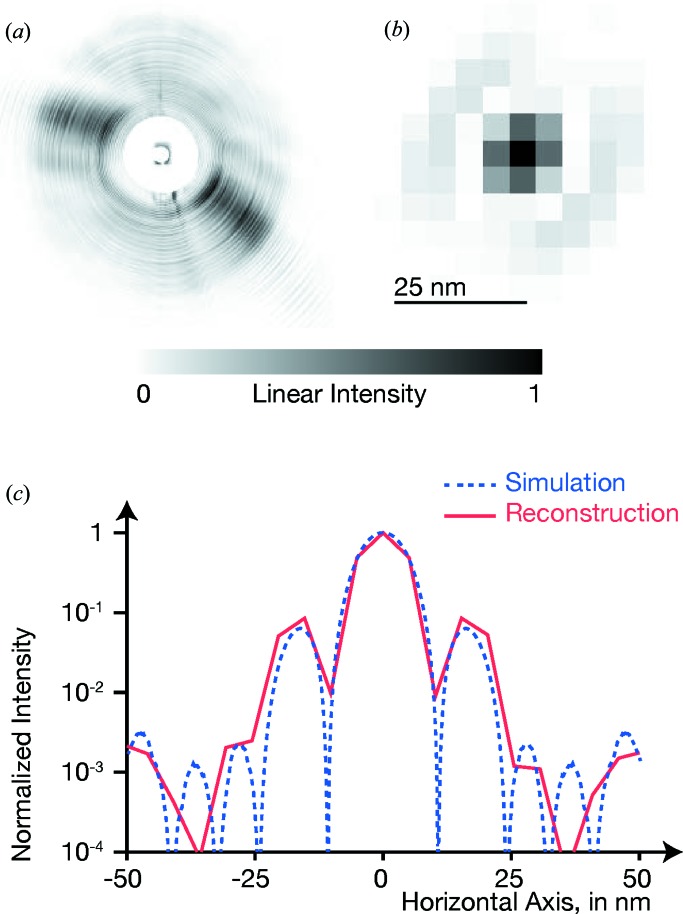
From far-field intensity patterns (*a*), a three-plane phase-reconstruction algorithm has calculated the intensity (*b*) (and phase, not shown here) in the +1st-order focus. (*c*) The horizontal line profile of the reconstruction (solid red line) is in agreement with the simulation (shown as a dashed blue line).

Flat phases and the measured intensity in 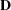 were used as initialization (see Fig. 2 ▶ *a*). The detector was illuminated in total for 200 s, with a vertical movement to stitch insensitive pixels and inter-module gaps. The three-plane phase-reconstruction algorithm is looped for 50 iterations, yielding the reconstructed focal intensity shown in Fig. 2 ▶(*b*). The horizontal line profile compares well to the simulation, as shown in Fig. 2 ▶(*c*). The focal spot size can be estimated to be close to the simulated value of 9.7 nm (FWHM).

### 2.5. Ptychography   

Ptychographic reconstructions solve the phase problem using constraints from scanning an object through the beam with significant overlap of the illuminated spots (Rodenburg *et al.*, 2007 ▶). This even allows for the simultaneous reconstruction of complex valued illumination (probe) (Hönig *et al.*, 2011 ▶; Kewish *et al.*, 2010 ▶; Thibault *et al.*, 2008 ▶) and transmission functions (object), assuming that the probe field does not change during the scan. To establish overlap in the case of nanometre-sized beams, such scans can be carried out in the defocus; the field can then be propagated numerically to the focal plane (Giewekemeyer *et al.*, 2013 ▶; Stockmar *et al.*, 2013 ▶; Wilke *et al.*, 2012 ▶).

In recent years, ptychography has been established as a standard tool to study both illuminations and optical systems, as well as different samples. The influence of beam stability, detector efficiency and noise characteristics, flux, and fluence requirements on ptychographic reconstruction accuracy, convergence and resolution are now well understood (Tripathi *et al.*, 2014 ▶; Schropp *et al.*, 2012 ▶; Maiden & Rodenburg, 2009 ▶), although in special cases spurious artefacts have been observed (Burdet *et al.*, 2014 ▶).

Ptychography has to meet stringent requirements, concerning in particular the step size to meet the necessary overlap as well as the detector pixel size and number. The used detector with pixel size 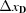 = 172 µm gives a field of view 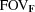 in the focal plane of 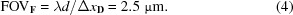
This does not capture the diverging negative order, which is about 6.0 µm in size at the +1st-order focal plane.

Ptychographic scans have been measured in several planes along the optical axis. While the algorithm does reconstruct the object (parts of a Siemens star), the probe fields are inconsistent. Disregarding the defocus position, the algorithm converges to delta peaks in all planes.

**Experimental issues**

Different shortcomings of the experiment have been identified. First, the available detector yields a field of view in the +1st-order focal plane of 2.5 µm, which does not capture the diverged −1st order or higher orders. Second, the virtual pixel size in this focal plane is about 5 nm as determined from the detector’s far-field coverage; hence, superresolution showing features below the nominal beam size of 10 nm cannot be resolved. Furthermore, the images are corrupted by vibrations and partial coherence, and the central part is distorted by the beamstop.

**Suitable parameters**

Successful reconstructions can be expected if the following experimental needs are met: The detector pixel size has to be smaller in order to extend the field of view around the diverging −1st order; for the given parameters, the pixels have to be smaller than 55 µm [*i.e.* not larger than the Maxipix or Medipix3 (*e.g.* LAMBDA) chip; Pennicard *et al.*, 2012 ▶]. On the other hand, the number of pixels then should to be accordingly higher, since the near-field sampling depends on the extent of the detector. Drift has to be measured, and vibrations should be smaller than 5 nm r.m.s. or accounted for by using illumination times shorter than the vibrational frequency and by applying drift correction.

## 3. Holographic imaging   

### 3.1. Tungsten line aperture   

To demonstrate the feasibility of holographic image acquisition with a multi-focus MZP, a W line aperture with a 1 µm-wide gap was positioned in the divergent beam of the −1st order. The optical thickness of the W is 3 µm, yielding a transmission of 62.3% and a phase shift of 2.704 rad.

**Figure 3:**
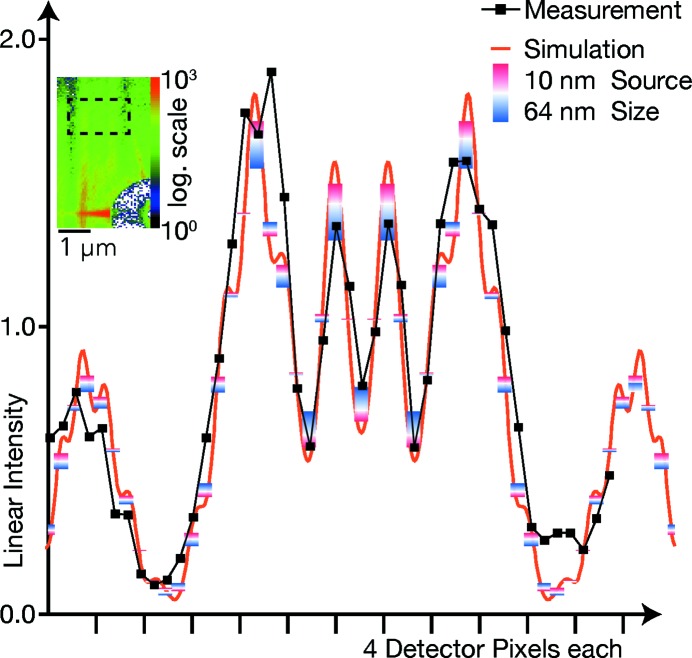
Measured and simulated Fresnel diffraction at a W bar aperture. In the inset, a typical detector image is shown with logarithmic intensity scale. One hundred images were correlated and added up, and then the depicted region of interest (dashed rectangle) was averaged vertically, giving the black squares in the graph. The red curve is a simulation for an ideal point source; the red-to-blue bars show the simulated intensity averaged over the virtual pixel size, for source sizes of 10–64 nm.

When the aperture is placed in the nominal focal plane of the +1st order, the defocus distance to the −1st focus is 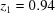
 mm. Together with the detector distance 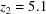
 m, this yields a magnification factor 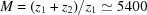 and a virtual pixel size of about 32 nm. A typical detector image is shown as an inset in Fig. 3 ▶; a characteristic fringe pattern due to Fresnel diffraction of the W aperture is clearly visible in the colour-coded intensity. To overcome drift, 100 images with exposure times of 10 ms each were correlated in position and then summed on a pixel-fine grid. After vertical summation of the region of interest (ROI, dashed rectangle), we obtain the horizontal line profile shown by black solid squares in Fig. 3 ▶.

In comparison, the red line shows the expected intensity profile 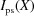 of the W aperture at the nominal distance from a point source, as modelled by the Fresnel cosine and sine functions (Cornu spiral), given by 
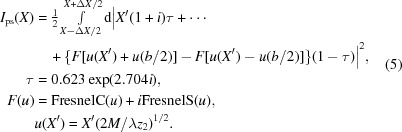



Here, 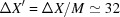
 nm is the demagnified pixel size. The constant term corresponds to absorption and phase shift inside the W foil, while the terms FresnelC(*u*) and FresnelS(*u*) model edge diffraction at both sides of the slit with width 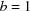
 µm. Varying the defocus distance, 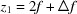, the Fresnel pattern is in good agreement for 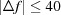
 µm. The W aperture was scanned through the X-ray beam; from the known velocity, the virtual pixel size of the hologram can be estimated to be 32 nm, in good agreement with the measured defocus distance.

In addition to the finite pixel size, also a finite source size σ with hat-top profile has been included. The intensity 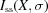 is defined by 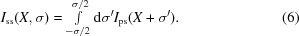
The corresponding data are shown by the coloured boxes in Fig. 3 ▶; the red end corresponds to the nominal source size of 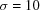
 nm, while the blue end models stronger vibrations and incoherence for 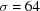
 nm. From the positions of the measured black squares, the effective source size is estimated to be around 50 nm; this enlargement can mainly be attributed to vibrations in the setup.

As expected, a larger source washes the fringe pattern out. Qualitatively, the measurement values (black squares) show a fringe contrast, indicating a resolution of better than 64 nm.

From these two observations, we conclude that holographic image acquisition in the diverging −1st order of the MZP is possible. Note that the third and higher orders diffract to larger angles on the detector and can be easily excluded from the data analysis. A similar experiment was carried out in a 1 mm defocus of the +1st order. See the supporting information for this data set.

### 3.2. GaAs nanowires   

On the sound footing that holographic data can be recorded with the −1st-order focus, we have advanced to holographic image acquisitions of individual semiconductor core–shell nanowires (NWs), with a core of GaAs and a shell of GaInP (Jacobsson *et al.*, 2012 ▶). The NWs were grown from nanoimprint lithographically defined Au particles from GaAs(111) with an SiN passivating mask between the particles [similar to the approach of Munshi *et al.* (2014 ▶), but with SiN instead of SiO and with Au evaporation before resist stripping]. For the X-ray imaging experiments, nanowires were mechanically transferred from the growth substrate to an X-ray-transparent Si
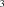
N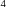 window using cleanroom tissue.

**Figure 4:**
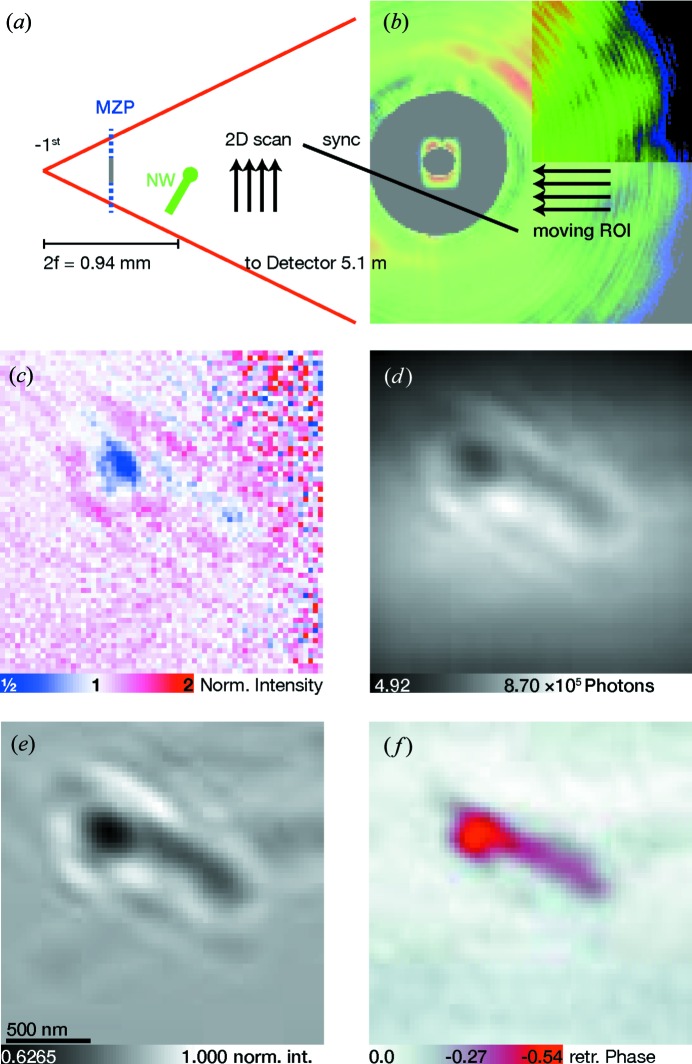
Holographic imaging of a nanowire. In (*a*), the illumination by the −1st-order MZP beam and the two-dimensional raster scan is shown. A typical detector image is shown in (*b*); the highlighted region of interest is a co-moving ROI linked to the raster scan. Part (*c*) shows a flat-field-divided ROI with a large noise floor due to the short illumination time. The image in (*d*) shows the correlated average of 1647 illuminations, flat-field corrected in (*e*). From this, the phase can be reconstructed by holography and a phase-retrieval algorithm, with the result shown in (*f*).

The two-dimensional scan experiment is sketched in Fig. 4 ▶(*a*). Part of a typical detector image is shown in Fig. 4 ▶(*b*); the highlighted ROI is moved synchronously to the nanowire to assist empty beam correction. Fig. 4 ▶(*c*) shows a single intensity image divided by an empty image without sample, on a logarithmic scale. Note that signal quality is affected by noise and artefacts from the highly structured MZP far-field pattern.

To overcome the noise artefacts, 1647 images of a co-moving ROI were added up (see Fig. 4 ▶ *d*) and then divided by a corresponding summation of empty images. The result is shown in Fig. 4 ▶(*e*). The signal-to-noise ratio is now sufficiently high. For the holographic setup, the demagnified pixel size is about 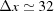
 nm, and 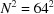 pixels were used. Qualitatively, only a few Fresnel fringes can be observed. This may be attributed to partial coherence and vibration effects. Using a holographic reconstruction, both amplitude and phase (complex transmission of the object) could be obtained, but this signal is distorted by a twin image. For phase reconstruction, the Gerchberg–Saxton algorithm has been employed. The resulting object phase is shown in Fig. 4 ▶(*f*).

For initialization, the far-field amplitude ψ with random phase per pixel and equal distribution in the interval 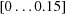
 rad is Fresnel propagated in equivalent geometry to the defocus plane (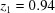
 mm, 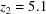
 m, Fresnel number 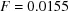), according to 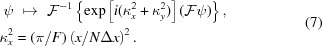



The nominal phase shift of a single nanowire is around 0.15 rad (400 nm of InP at 18 keV), and the absorption is 0.006; the nanowire’s ‘head’ is roughly twice as thick. The low absorption allows us to use the ‘pure phase object’ constraint in the sample plane as *a priori* knowledge: The reconstructed phase is kept, but the amplitude (intensity) is set to unity. The updated object is propagated to the detector, where again the phase is kept and the intensity is re-set now to the measured values.

The number of iterations (100–10 000) seems to have little influence. By far the most critical parameter is the defocus distance 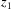 (or the Fresnel number *F*). During alignment, this was controlled using an optical microscope, measuring the distance of the sample from the MZP plane.

The empty beam corrected hologram and the resulting reconstruction show that, despite the very structured multi-mode illumination, holographic recordings are possible with the MZP. However, significant deficits in image quality persist. In addition, the validity of the empty beam correction can be questioned (Hagemann *et al.*, 2014 ▶).

## 4. Holographic STXM   

Next, we present a first attempt to image a test sample in STXM mode. A Siemens star is scanned through the focal plane and a detector records intensities 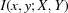; the coordinates 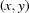 are the sample’s position, and 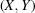 are the detector coordinates (Thibault *et al.*, 2008 ▶). We generalize from measurements in the focal plane to measurements at a defocus distance 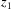. As scalar observable, we use the horizontal centre-of-mass 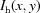, 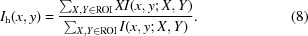
In the focal plane, 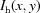 is related to the differential phase contrast (Menzel *et al.*, 2010 ▶; Thibault *et al.*, 2009 ▶; de Jonge *et al.*, 2008 ▶; Morrison & Niemann, 1998 ▶) in the horizontal direction, convoluted with the focused beam. The same applies for the vertical direction.

**Figure 5:**
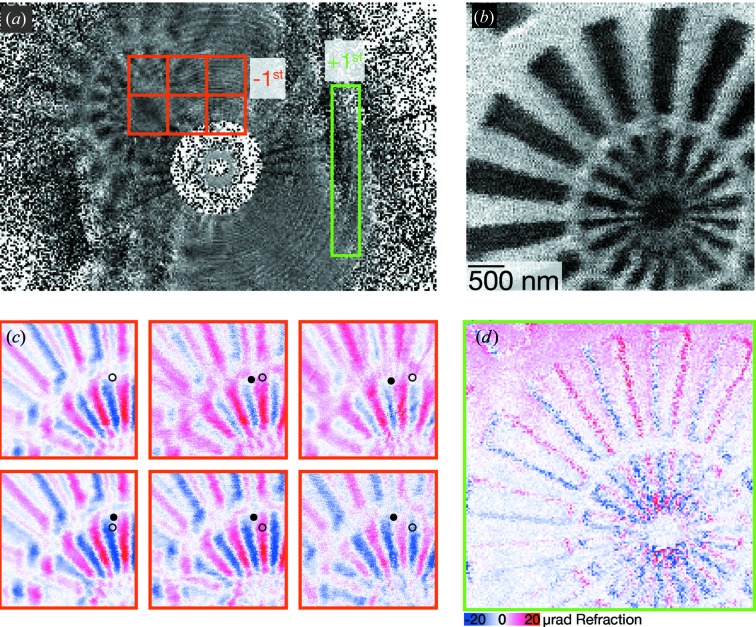
Holographic STXM. (*a*) A single holographic image of the Siemens star, illuminated by the −1st-order focus. (*b*) The horizontal centre-of-mass STXM image if the full detector is used. The images in (*c*) correspond to the different orange ROIs, while (*d*) corresponds to the green ROI. The blue-to-red colour map quantifies the refraction angle proportional to differential phase contrast. For a discussion of the circles in (*c*), see the text. The STXM scan consists of 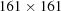 images, each illuminated for 10 ms, and a field of view of 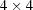
 µm.

A Siemens star test pattern (500 nm Au on an Si
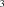
N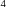 membrane) with smallest feature sizes of 50 nm was placed in the nominal +1st-order focal plane. Again, holograms emerging from the −1st order are visible on the detector images, facilitating the alignment because of their large field of view. A single detector image after empty beam division is shown in Fig. 5 ▶(*a*); a hologram of the Siemens star is visible in the top left region. Continuous raster scans of 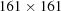 points over a field of view of 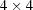
 µm, with illumination times of 10 ms per point, have been recorded. See the supporting information for this data set and a small analysis programme.

Taking the whole detector image into account in evaluating equation (3) yields the STXM map shown in Fig. 5 ▶(*b*). The 50 nm features are hardly visible. It should be noted that this STXM map does not show the usual horizontal edges, as is expected for differential phase contrast.

Taking only the ‘+1st-order’ ROI, indicated by the green box in Fig. 5 ▶(*a*), for the calculation of equation (8) yields the STXM map shown in Fig. 5 ▶(*d*). Horizontal edges can be clearly seen, indicated by the blue and red colours; vertical edges are not visible in the horizontal phase contrast. This signal can be attributed to the usual STXM contrast as carried out in the focal plane.

Next, we address the question of whether the −1st order can also be used for STXM. For a defocus distance 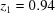
 mm, this component of the MZP beam has a diameter of more than 6 µm. Following the usual approximations (Menzel *et al.*, 2010 ▶), a STXM measurement with such a large beam should not give any result. But the six STXM maps shown in Fig. 5 ▶(*c*) show the differential phase contrast corresponding to the ROIs marked in orange in part (*a*). Clearly, a holographic signal is visible. The inset open circle is drawn at the same coordinates, while the filled circle follows a specific feature of the Siemens star, as the ROI is moved over the detector. This movement is in agreement with a demagnified pixel size of 32 nm and hence 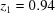
 mm. These STXM maps thus are images by the −1st order, taken with a beam size larger than the scan size.

Next we investigate for which conditions this holo-STXM scheme can be used and what resolution can be achieved. A one-dimensional sample is described by its complex valued transmission function 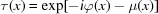 with (smooth) phase shift 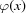 and absorption 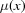, which we neglect in the following. With a small X-ray beam centred around position 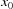, the phase can be expressed by a Taylor expansion 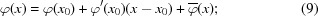
if the higher-order residuals 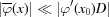, these can be neglected, and equation (8) effectively quantifies the 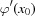 contribution. Let us assume that the phase shift 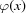 is taken of a ‘limited’ Schwartz space, 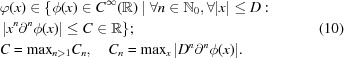
Then the *smoothness parameter C* is an upper bound on all derivatives over the length scale *D* of the beam size. The Taylor residuals are bounded by 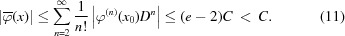
If now 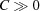, the higher-order contributions 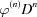 cannot be neglected in general and will contribute to equation (8). Then, STXM with a 6 µm large beam should not give the maps shown in Fig. 5 ▶(*c*), where 100 nm features are visible.

**Figure 6:**
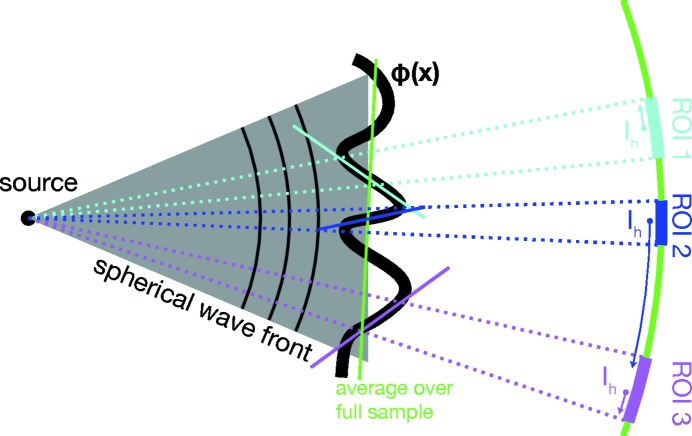
Although the illuminating beam is larger than the sample, the local structure can be resolved if the curved wavefronts fall onto separated regions on the detector.

On the other hand, the sample is illuminated by a diverging wavefront. As illustrated in Fig. 6 ▶, the STXM contrast over small ROIs on the detector contains local information about the sample, although the illuminating beam may be much larger.

As shown in ROI 3 in Fig. 6 ▶, this argument is not applicable for very large *C*, when parts of the sample scatter too far into neighbouring ROIs on the detector. In the example, the sharp blue edge in the sample spoils the centre-of-mass 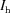 of ROI 3. Assume ROIs with *N* pixels of virtual (demagnified) pixel size 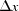 within a distance 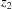. To first order, the centre-of-mass is shifted by 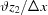 pixels, with the refraction angle 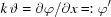. Not to spoil other ROIs, the refraction needs to be small compared to the ROI size, 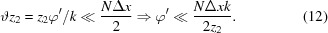
But for large *N*, too much structure variation is averaged out. We found 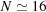, yielding good signal-to-noise ratio and structure information. With the parameters of this experiment, phase gradients 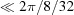
 nm can be measured.

## 5. Conclusion and outlook   

We have shown that hard X-ray imaging can be carried out in a multi-order setup using a focusing MZP, without an order-sorting aperture. Using a three-plane phase reconstruction, the focus size of the MZP has been estimated to match the simulated size of less than 10 nm. From fitting analysis of holograms, the virtual source size of the diverging −1st order, including broadening due to vibration and partial coherence, has been estimated to be approximately 50 nm, in agreement with the holo-STXM data. Iterative phase retrieval has been used to image individual GaAs/GaInP nanowires. Further, we have generalized scanning transmission X-ray microscopy, where the sample is scanned in the focal plane, to a holo-STXM experiment carried out in the defocus. This allows us to resolve details in the scanned sample, even if the illuminating beam is much larger than the sample or scan region, as is shown in our theoretical treatment. The simultaneous illumination with the diverging −1st order may allow for low-resolution images with reduced dose for the purpose of alignment of delicate biological samples. This multi-order technique is proposed as a new imaging method in the ‘zoo’ of X-ray imaging methods, with both assets and drawbacks of its own.

The experiments show that the order-sorting aperture can in principle be replaced during image processing and analysis, employing a ‘software OSA’. The actual performance of this imaging methodology is to be further investigated. Experiments with real-time measurements of the sample’s position relative to the MZP with an interferometric system currently under commissioning are planned, and algorithms including this information are being developed. To meet the demanding stability requirements for beams of a few nanometres in size, a dedicated setup with a short distance between the MZP and sample is developed. We expect imaging resolution comparable to the current 5–10 nm resolution obtained with ptychography, but now also for other relevant contrast mechanisms, *e.g.* nanodiffraction, fluorescence mapping and local excitation.

## Supplementary Material

Supporting information file. DOI: 10.1107/S1600576714026016/to5096sup1.pdf

Supporting information file. DOI: 10.1107/S1600576714026016/to5096sup2.bin

Supporting information file. DOI: 10.1107/S1600576714026016/to5096sup3.txt

Supporting information file. DOI: 10.1107/S1600576714026016/to5096sup4.txt

## Footnotes

1 Supporting information for this paper is available from the IUCr electronic archives (Reference: TO5096).
