# Supplementary material for: Towards multi-order hard X-ray imaging with multilayer zone plates
Source: J Appl Crystallogr. 2015 Jan 30;48(Pt 1):116–24. doi: 10.1107/S1600576714026016 (PMC4453173; doi:10.1107/S1600576714026016)
Supplement: Supplementary file 1 [file j-48-00116-sup1.pdf]

Journal of Applied Crystallography

ISSN 0021-8898

Supplemental Material to: Towards multi-order hard x-ray imaging with multilayer zone plates

Markus Osterhoff,[a]* Christian Eberl,[b] Florian Döring,[b] Robin N. Wilke,[a] Jesper Wallentin,[a] Hans-Ulrich Krebs,[b] Michael Sprung[c] and Tim Salditt[a]

[a]Institut für Röntgenphysik, Georg-August-Universität Göttingen, Friedrich-Hund-Platz 1, 37077 Göttingen, Germany, [b]Institut für Materialphysik, Georg-August-Universität Göttingen, Friedrich-Hund-Platz 1, 37077 Göttingen, Germany, and [c]DESY Photon Science, Notkestraße 85, 22607 Hamburg, Germany. Correspondence e-mail: mosterh1@gwdg.de

© 2014 International Union of Crystallography
Printed in Singapore – all rights reserved

This Supplemental Material to our paper entitled "Towards multi-order hard x-ray imaging with multilayer zone plates" makes two central files of raw data available. In order to inspire discussions about the proposed imaging scheme of holographic scanning microscopy (holo-STXM), an analysis code is included. Additionally, a video of one holographic scan is published to visualise simultaneous imaging in two diffractive orders of the MZP illumination.

1. Description of datasets

The two dataset files, `lineaperture.h5` and `holosiemens.h5`, contain the raw data (central module of Pilatus 300k detector only) in HDF 5 format. First we describe the HDF 5 layout and metadata structure; in the following subsections, the respective experiments are described and all relevant parameters are stated. In section 2, the holo-STXM experiment and the analysis code is described. See Fig. 1 for a sketch of the experiments and relevant parameters.

1.1. File format

Within the HDF 5 container, the data is stored as matrices. Detector data is saved as integer values (unsigned integer, 16 bit) representing single photon counts per pixel; motor positions are encoded as floating point numbers in metre. For details, cf. Tab. 1. Fig. 2 visualises the layout of the three- and four-dimensional data as 2D images taken at 1D and 2D sample positions.

1.2. Tungsten line apertures in holography

The dataset `lineaperture.h5` contains raw Pilatus 300k images (central module), taken during a horizontal scan of a vertical W line aperture test pattern. The sample was mounted on a piezo stage and scanned by 100 µm in 200 steps, with an accumulation time of 100 ms per point. Encoder values of the piezo axis are also available in the file. The experimental setup is similar to that of Fig. 3 in the main paper, but with the sample placed 1 mm in the defocus of the +1st focusing order of the MZP, and in a defocus of 2 mm of the coexistent -1st order.

If the images are played as a video (see file `lineaperture.mp4`), the vertical lines of the test sample move from the right side to the left side. As can be seen, holographic images on the right part of the detector are much wider and faster than those on the left part. This is attributed to the different focusing orders: Due to a slight detuning of the MZP angles, the +1st order produces holographic images on the right side of the detector, and -1st order produces images on the left side. The corresponding virtual pixel sizes are

$$\Delta x_+ \approx 32 \text{ nm}, \quad \Delta x_- \approx 64 \text{ nm},$$

which can be calculated by the Fresnel scaling theorem with magnifications

$$M = \frac{z_1 + z_2}{z_1}, \quad M_+ \approx 5100\times, \quad M_- \approx 2050\times,$$

for $z_{1,+} = 1$ mm, $z_{1,-} = 2$ mm, $z_2 = 5.1$ m and a Pilatus pixel size of 172 µm. These pixel sizes are in agreement with the velocity of the holographic images on the detector and the actual real-space movement of the piezo. From this comparison it is concluded that the holographic images in fact are due to the respective focusing orders of the MZP.

1.3. Video file of this scan

The file `lineaperture.mp4` contains a video of detector images taken during a horizontal scan of the W line aperture sample ("barcode" of several apertures with varying width and distance). The intensity is shown on a logarithmic scale; for the colour bar, cf. Fig. 2. The +1st and -1st diffraction orders of the MZP fill the greenish disk, approx. 200 pixel in diameter. In the centre, the primary beam has been blocked by beam stops. The line apertures are scanned horizontally from right to left, in a defocus of 1 mm downstream of the +1st order focal plane, and accordingly 2 mm downstrean of the -1st order virtual focus. The corresponding pixel sizes are $\Delta x_+ = 64$ nm and $\Delta x_- = 32$ nm; the step size of the scan is 500 nm with an illumination time of 100 ms. The video is encoded at 25 fps, so it plays at 2.5× nominal speed. In the video, vertical patterns move from the right to the left. These are holographic images from the line aperture. Note that due to a detuning of the MZP, the right hand side (RHS) of the detector is mainly illuminated by the +1st order, while the left hand side (LHS) is mainly illuminated by the -1st order of the MZP. As can be seen, the lines in the different

file	path	contains	format
lineaperture.h5	pilatus	raw data from Pilatus 300k central module	$201 \times 487 \times 195$ integers
lineaperture.h5	positions	motor positions (encoder values) of piezo	201 double values, in metre
holosiemens.h5	pilatus	raw data from Pilatus 300k central module	$161 \times 161 \times 487 \times 195$ integers
holosiemens.h5	positions	motor positions (encoder values) of piezo	$161 \times 161 \times 2$ double values, in metre

Table 1: Layout of the HDF 5 containers storing detector image data and motor positions.

parts have different sizes and velocities. An analysis shows on the RHS, the lines are twice as large and twice as fast. The hologram sizes and velocities are in agreement with the two pixel sizes.

1.4. Siemens star in holo-STXM

The second dataset, stored in file `holosiemens.h5`, contains the Pilatus images of a STXM scan. A Siemens star test pattern (500 nm Au on a Si_3N_4 membrane) has been scanned in two dimensions, with a scan range of 4×4 µm^2 and 160×160 steps in both directions. The accumulation time was 10 ms, at a constant movement of the horizontal piezo axis (continuous scan). The samples was placed close to the focal plane of the +1st MZP order, as determined by an optical microscope. The demagnified pixel size of the -1st order is 32 nm. Also contributions of the $\pm 3^{rd}$ focusing order can be identified, but with a very weak signal-to-noise ratio.

This dataset can be described as a four dimensional matrix $I(x,y;X,Y)$ with sample position (x,y) and detector pixels (X,Y). In the usual STXM sense, the horizontal centre-of-mass $I_h(x,y)$ as a function of sample position is defined as the first moment over the detector, or here over a region of interest (ROI):

$$I_h(x,y) = \frac{\sum_{X,Y \in \mathrm{ROI}} X \cdot I(x,y;X,Y)}{\sum_{X,Y \in \mathrm{ROI}} I(x,y;X,Y)}$$

In focal-plane STXM, this is related to the differential phase contrast $I_h(x,y) \propto \partial\varphi/\partial_x$ of the sample. We found that for specific ROIs, the images $I_h(x,y)$ show the typical behaviour with horizontal edge enhancement and the absence of vertical edges. Other ROIs, on the other hand, produce holographic images; the field of view moves if the ROI is changed, with a pixel "velocity" according to the demagnified pixel size of the -1st order. In the next section, we describe an analysis programme to calculate the images of Fig. 5 (c,d) in the main paper, using dataset `holosiemens.h5`.

2. Description of holo-STXM programme

To visualise the dataset `holosiemens.h5` in the described holographic STXM mode, the source codes `holosiemens.m` (Matlab) and `holosiemens.c` (ANSI C, needs HDF 5 library) are available. The Matlab script consists of three cells:

1. read in the HDF 5 data set (needs approx. 10 GB of memory);
2. select a region-of-interest on the detector; seven ROIs according to the green (0) and the orange ROIs (1-6) in Fig. 3 are pre-defined;
3. calculate and show the corresponding STXM $I_h(x,y)$ image.

With the ROI 0 selected, the STXM image resembles usual differential phase contrast features. Horizontal edges of the Siemens star pattern show alternating positive and negative values; vertical edges remain invisible. Regions of constant phase shift share a common gray value.

But ROIs 1...6 look qualitatively different. Instead of edge enhancement, a holographic image appears. Furthermore, the different ROIs show a different view of the Siemens star. From the feature size (smallest lines and stripes are 50 nm apart), the demagnified pixel size of ≈ 32 nm can be estimated. This correspond to a magnification factor $M \approx 5100$, and a defocus distance of $z_1 = 1$ mm, in full agreement to the hypothesis of a diverging -1st order illumination by the MZP.

In addition to the Matlab routine, also an ANSI C code programme `holosiemens.c` is available. Comments how to compile and use the code are included. It has been developed for `gcc 4`, but should run with all common compilers. The HDF 5 library is needed.

See Fig. 1 for further physical and geometrical parameters of the experiment.

3. Summary

With this Supplemental Material to our paper entitled "Towards multi-order hard x-ray imaging with multilayer zone plates", experimental raw data of two holographic imaging experiments is made available. To inspire analysis by the holo-STXM method, an analysis code is included. We shortly list the published files:

`lineaperture.h5`	raw data of the W line pattern scan
`lineaperture.mp4`	video created from the above
`holosiemens.h5`	raw data of the Siemens star STXM
`holosiemens.m`	Matlab routine to calculate $I_h(x,y)$
`holosiemens.c`	equivalent C code programme

Due to file size, `holosiemens.h5` can currently be downloaded at `http://www.roentgen.physik.uni-goettingen.de/~mosterh/holosiemens.h5`.

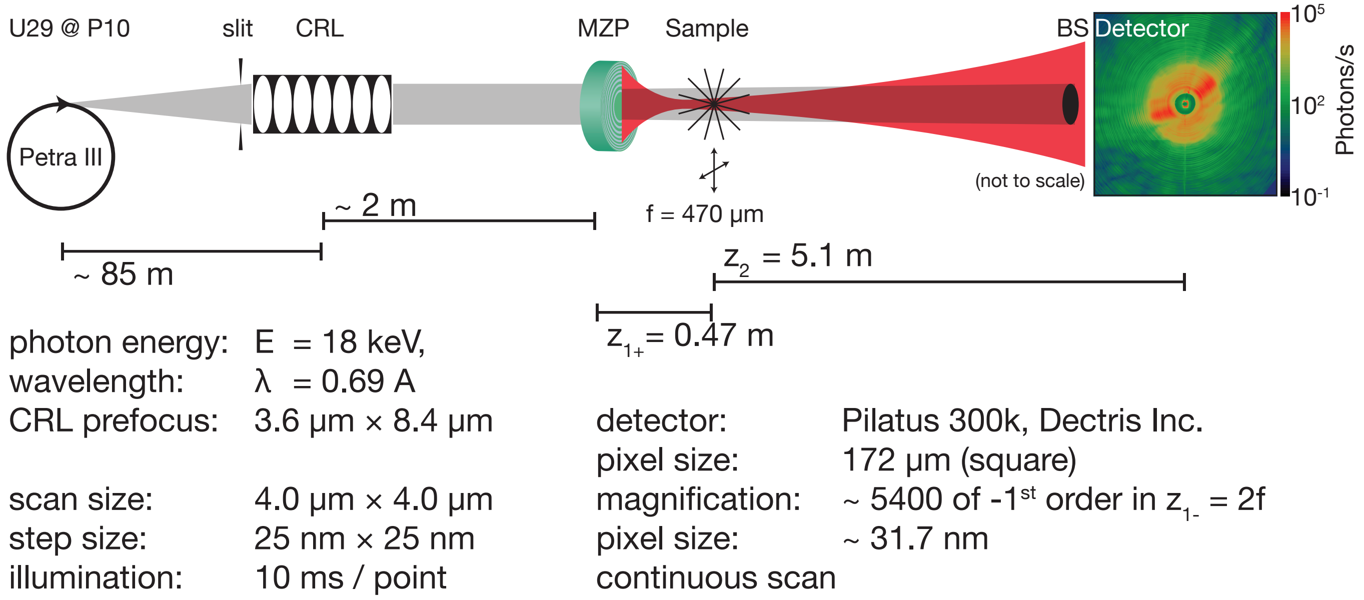

Figure 1: Geometrical and physical parameters of the holo-STXM experiment.

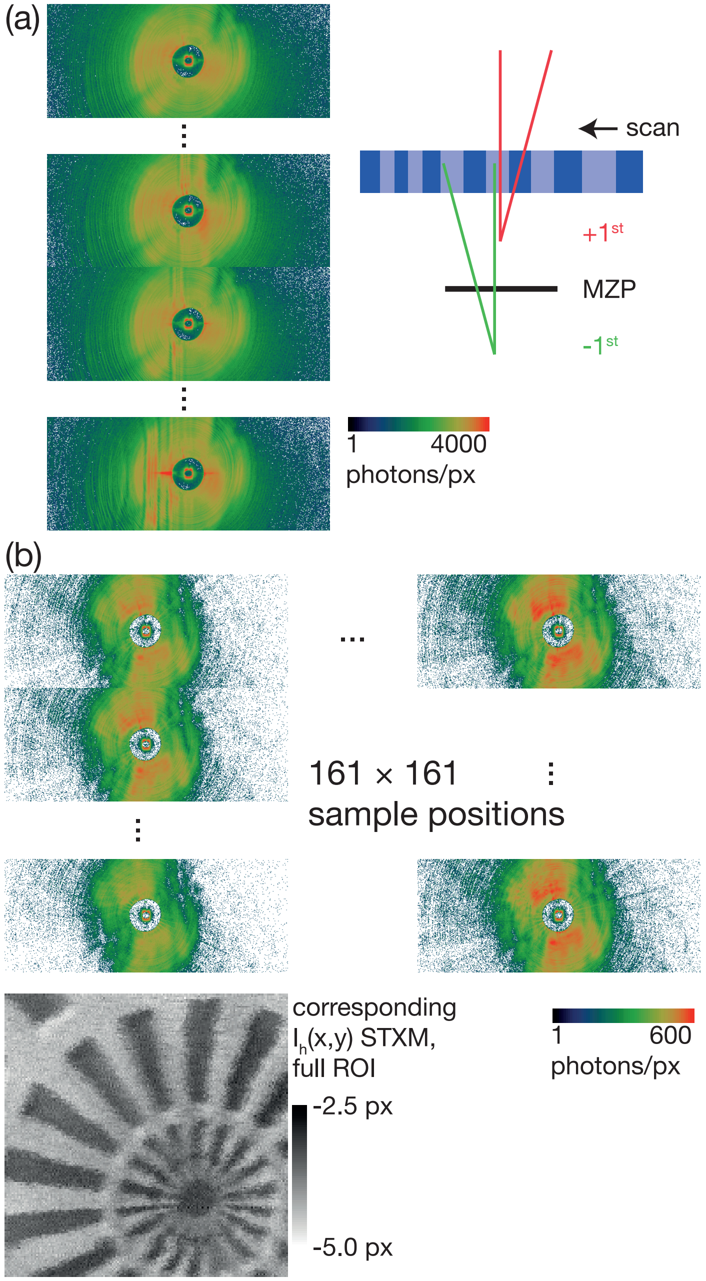

Figure 2: The layout of the datasets. (a) The file `lineaperture.h5` contains 2D detector images along a 1D scan of the sample. (b) The file `holosiemens.h5` contains 2D detector images, taken on a 2D grid of sample positions.
